# Circadian rhythmicity in schizophrenia male patients with and without substance use disorder comorbidity

**DOI:** 10.1007/s00406-023-01560-7

**Published:** 2023-03-06

**Authors:** Ana Adan, Julia E. Marquez-Arrico, Laura Río-Martínez, José Francisco Navarro, Antonio Martinez-Nicolas

**Affiliations:** 1 Department of Clinical Psychology and Psychobiology, School of Psychology, University of Barcelona, Passeig de la Vall d’Hebrón 171, 08035 Barcelona, Spain; 2 Institute of Neurosciences, University of Barcelona, 08035 Barcelona, Spain; 3 Department of Psychobiology, School of Psychology, University of Málaga, Campus de Teatinos s/n, 29071 Málaga, Spain; 4 Chronobiology Lab, Department of Physiology, College of Biology, University of Murcia, Mare Nostrum Campus, IUIE, IMIB-Arrixaca, 30100 Murcia, Spain; 5 Human Physiology Area, Faculty of Sport Sciences, University of Murcia, Santiago de La Ribera-San Javier, 30720 Murcia, Spain; 6 CIBER Fragilidad y Envejecimiento Saludable, Instituto de Salud Carlos III, 28029 Madrid, Spain

**Keywords:** Circadian rhythmicity, Schizophrenia, Dual schizophrenia, Dual disorders, Substance use disorders, Distal skin temperature

## Abstract

Circadian rhythmicity is associated to clinical variables that play an important role in both schizophrenia (SZ) and substance use disorders (SUD), although the characteristics of the coexistence of these two diagnoses (SZ +) remain mostly unknown. Hence, we studied a sample of 165 male patients divided in three groups each of 55, according to their diagnoses (SZ + , SZ, and SUD), as well as a healthy control (HC; *n* = 90) group. Alongside with sociodemographic and clinical variables, circadian rhythms were registered through a sleep–wake data structured interview, a circadian typology questionnaire, and distal skin temperature (DST) using the Thermochron iButton every 2 min during 48 h. Analyses showed that SZ + and SZ patients presented a longer sleep (delay in wake-up time) and mostly an intermediate circadian typology, while SUD patients slept less hours, displaying a morning typology. The DST showed the highest daily activation and stability for the SUD group, even when compared with the HC group. The presence of schizophrenia (SZ + and SZ) was related to a DST pattern with a reduced amplitude determined by a wakefulness impairment, which was more pronounced for SZ patients whose sleep period was adequate. The assessment of circadian rhythms in under treatment male patients with SZ should be focused on the diurnal period as a possible marker of either treatment adherence or patient's recovery, irrespective of the presence of a comorbid SUD. Further research with additional objective measures may provide knowledge transferable to therapeutic strategies and could be useful to establish possible endophenotypes in the future.

## Introduction

Substance use disorders (SUD) are a global public health problem, that require treatment and in which a clear difference between the sexes is observed, with the admissions of men accounting for around three quarters [1]. SUD is especially prevailing in patients with schizophrenia (SZ); prevalence rates of SUD in patients with psychotic spectrum disorders are around 50% [2]. This comorbidity, termed dual schizophrenia (SZ +), is also more frequent in men [3] and linked to worse sociodemographic profile and clinical severity [4]. In patients with SZ + , poorer therapeutic results and less adherence to treatment was observed [5], as well as more suicide attempts [6] and an earlier onset of SUD and SZ [7] compared to those with single diagnosis.

Circadian rhythmicity has clinical and social relevance [8], with variability in its expression among individuals. Circadian typology is based on differences in the phase of circadian rhythmicity (morning-, intermediate-, and evening-type) and is related to health habits and lifestyles [9]. Evening-type is associated with less healthy habits and is considered a risk factor for developing mental disorders [10]. The distal skin temperature (DST) rhythm is an indirect measure of the functioning of biological clocks, postulated as a marker rhythm due to its ease of measurement in the consensus document “Developing Biomarker Arrays Predicting Sleep and Circadian-Coupled Risks to Health” that was sponsored by the US Department of Health & Human Services [11]. DST measurement has shown validity in detecting the sleep–wake [12] cycle, ability to discern among the three circadian typologies [13] and its stable phase relationship with melatonin secretion pattern [14].

Alterations in circadian rhythms have been observed in patients with SUD even after months of abstinence, being the most frequent a phase delay (eveningness) and a reduction in amplitude [15]. These patients complain about the quality of their sleep and sleep disturbances which do not always respond to medication [3]. In this sense, imposing stable schedules during SUD treatment is a positive factor in the recovery of circadian rhythmicity, which is better in residential vs. outpatient treatment [16].

Patients with SZ also exhibit sleep–wake rhythm disturbances associated with a worse clinical status and poorer therapeutic outcomes [17, 18]. These disturbances are present before the first psychotic episode with insomnia in up to 50% of the cases [19] along with higher rates of hypersomnia than in healthy controls and bipolar disorder patients [17]. Insomnia would exacerbate psychotic symptomatology and vice versa [20]. The few studies evaluating circadian rhythmicity in SZ point to an alteration in its regulation [21, 22] that could be related to poor endogenous control (i.e. melatonin secretion) and to lifestyle habits that hinder adequate exposure to external synchronizers [23]. Furthermore, the presence of evening typology in SZ is higher than in control individuals and is three times higher than in bipolar disorder [17], being associated with greater sleep phase delay and irregularity, which in turn is related to a worse clinical condition [23]. Sleep disturbances and circadian rhythmicity were analyzed as a possible endophenotype for SZ [24] and, in this line, our study is a novel contribution.

Finally, in the only study, we found on circadian rhythmicity in SZ + , assessed by DST, a greater affectation is observed with respect to patients with comorbid depression and bipolar disorder [25]. Other studies focus on sleep and indicate that a SUD aggravates the reduction of sleep duration and quality, and increases the prevalence of insomnia, affecting psychotic symptomatology [17, 26]. The existence of possible alterations in SZ + is frequently extrapolated from studies in patients with only SUD and/or only SZ [27]. In such cases, the rate of sleep disturbances is higher in patients with SZ and history of SUD [17].

Our work addresses, for the first time, possible differences in the circadian rhythm of SZ patients with/without a diagnosis of SUD (SZ + , SZ) compared to patients with only SUD and to healthy controls (HC). The circadian rhythm was assessed with self-assessed sleep parameters and circadian typology, and with DST rhythm recording which provides comprehensive information on the circadian system and the quality of wakefulness.

## Methods

### Participants and study design

**Fig. 1:**
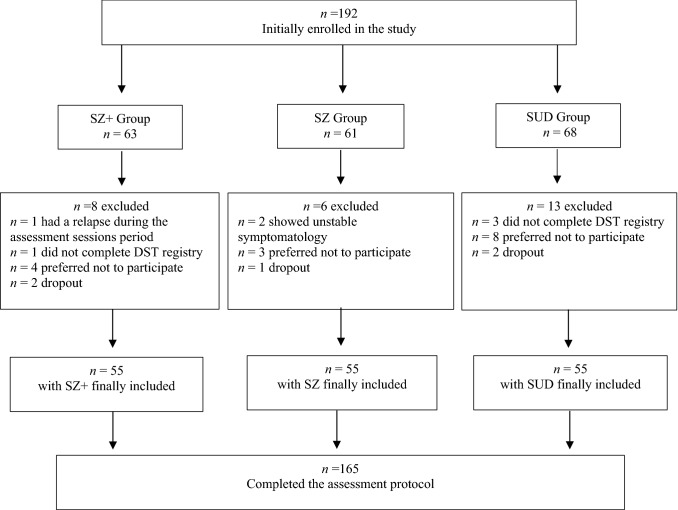
Study sample flow diagram. *SZ* + dual schizophrenia, *SZ* schizophrenia only, *SUD* substance use disorder, *DST* distal skin temperature

A final total sample of *N* = 165 under treatment patients in different public/private centers in the province of Barcelona (Catalonia), were assigned to one of the three groups (each of *N* = 55) according to their diagnosis (see Fig. 1). The inclusion criteria were: (1) male sex because of the higher prevalence rates of SZ and SUD in males as well as to control the possible differences in the clinical manifestations of both SZ and SUD between men and women; (2) aged 18–55, excluding adolescents and older people for being a minority in treatment centers and with differential rhythmic aspects associated with age; (3) under treatment and stabilized; (4) with a SUD diagnosis in initial remission for SUD and SZ + groups, according to Diagnostic and Statistical Manual of Mental Disorders (DSM-5) criteria [26]; (5) with a diagnosis of schizophrenia for the SZ and SZ + groups, according to DSM-5 criteria. In the groups with SUD, abstinence was verified by urinalysis. The exclusion criteria were presenting with induced disorders, not yet with stabilized symptoms or in remission, and any condition that affected the assessments process (i.e. neurological and neurocognitive disorders).

We included a HC group (*N* = 90 males) whose epidemiology and DST data were provided by Chronobiology Lab (University of Murcia). These participants met the same inclusion and exclusion criteria, adding that they must not present any current/past DSM-5 diagnosis.

### Procedure

Patients were referred to our research from their treatment centers by their professionals based on inclusion/exclusion criteria. We contacted each participant and they were provided and signed an informed consent, participants were not compensated for their participation. The study protocol, which is part of a wider research project, was administered during approximately 2 h individually for each participant by a postgraduate psychologist at their treatment center. The protocol administration order was as follows: sociodemographic, clinical and circadian assessment, and placement of Thermochron iButton^®^ which was collected after 48 h of registration.

### Sociodemographic and clinical assessment

We used a structured interview for collecting sociodemographic and clinical variables. We converted the dose of antipsychotic drugs to milligrams of chlorpromazine. Diagnoses were confirmed and detailed with the Structured Clinical Interview (SCID-I) for DSM-IV-TR [28]. The Spanish version of Global Assessment of Functioning (GAF) scale was administered to assess general functioning [28]. The validated Spanish version of Drug Abuse Screening Test [29] (DAST-20) was applied for measuring severity of addiction in the SUD and SZ + groups, since it has shown very good psychometric properties [29]. The DAST-20 provides a value for severity of addiction following these cut-off points: 0 no addiction, 1–5 mild, 6–10 intermediate, 11–15 high, and 16–20 severe [29]. For assessing psychotic symptoms in SZ and SZ + groups, we used the Spanish version of the Positive and Negative Syndrome Scale (PANSS) [30] which has demonstrated to be an adequate instrument for this population [30].

### Circadian rhythms assessment

We collected sleep–wake data with a structured interview specifically designed; we asked for habitual wake-up time, bedtime, total time sleeping, and absence/presence of nap and its duration.

To assess circadian typology, we administered the Composite Scale of Morningness (CSM) in its Spanish version [31] with an adequate internal consistency in our total sample of patients (Cronbach´s alpha = 0.845). The CSM gives a score which classifies circadian typology as follows: 0–25 evening-type, 26–36 neither-type, and scores from 37 morning-type.

We registered DST every 2 min for 48 h using the Thermochron iButton^®^ DS1921H device (*Maxim Integrated Products*, Sunnyvale, California, USA). The sensor was placed in the wrist of the non-dominant hand, over the radial artery, to reduce the potential masking effects generated by the higher activity of the dominant hand [32]. We carried out two types of analyses by the Circadianware^™^ software version 7.1.1 [33] for obtaining several parameters [34]. First, we obtained minimum, maximum, mesor, acrophase, Rayleigh vector, Fourier analysis with the first 12 harmonics, circadianity index [CI; calculated as first harmonic power (P1)/sum of 12th harmonics power (P12)], a measure of relative power of the circadian component compared with the ultradian components, and maximum and minimum temperature with cosinor analysis (parametric). Second, we conducted a nonparametric analysis and calculated interdaily stability (IS), intradaily variability (IV), relative amplitude multiplicated per 10 (RA_10), maximum mean temperature in 5 consecutive hours (M5) and its time (TM5), minimum mean temperature in 10 consecutive hours (L10) and its time (TL10). Two days of recording are sufficient for the reliability of all parametric and most nonparametric parameters. Thus, the nonparametric IS index requires a minimum of 3 days and we have followed the recommendation of Van Someren et al. [35] to eliminate from the analysis values > 1.

### Statistical analysis

Descriptive data (mean, standard deviation and percentages) were obtained for sociodemographic and clinical data. Group differences in these variables were explored with analysis of covariance (ANCOVA) for continuous data, and Kruskall–Wallis tests for non-continuous data. When the variables affected only two groups (data from SZ or SUD diagnoses), we applied the student´s *t*-test (*t*) if the quantitative data fulfilled the necessary conditions; otherwise, Mann–Whitney *U* test was used. Chi-Square contrast was applied for categorical variables.

We carried out an ANCOVA for CSM scores, and three multiple analysis of covariance (MANCOVA) considering sleep schedule, and nonparametric and parametric DST data as dependent variables. In all cases, we considered group as independent variable, and age as covariate since it could be a confounding factor [34, 36]. Post hoc comparisons were Bonferroni corrected to adjust the level of significance to the multiple comparisons and partial square Eta (*η*_*p*_^2^) or Cohen’s d were calculated to measure the effect size.

All data were analyzed using the SPSS 25.0 software (IMB Corp, Armonk, NY, USA). All tests were two-tailed (*p* < 0.05) and no data were excluded from the analyses since all the participants that were finally included in the sample completed all the assessment protocol.

## Results

### Sociodemographic and clinical variables

Table 1 presents sociodemographic data for the four groups, and clinical data for the three groups with a diagnostic. Mean age for the total sample was 37.30 ± 8.82 years old, without differences among groups. The HC group included a higher number of married or stable partner than the clinical groups. In the clinical groups, the majority of patients were single, although we found differences between the SZ and SUD groups (*p* = 0.013), with fewer people married or with a partner in the former case. On the other hand, in the HC group, there was a higher percentage of employed people compared to the clinical groups, in which the SUD group contributed more employed patients compared to the SZ and SZ + groups (*p* ≤ 0.010 in both cases). Finally, we found differences in the years of schooling (*F*_(3,251)_ = 25.13; *p* < 0.001; *ηp*^*2*^ = 0.26), with a higher level of education in the HC group compared to the clinical groups (*p* < 0.001 in all cases), which had not any differences among them.

**Table 1:** Sociodemographic data for the clinical and control groups. Means, standard deviation, percentages, and statistical contrasts

	SZ + (*n* = 55)	SZ (*n* = 55)	SUD (*n* = 55)	HC (*n* = 90)	Contrasts
Age (years)	36.00 ± 8.19	39.07 ± 8.72	35.78 ± 6.98	37.94 ± 10.04	*F*_(3,251)_ = 1.86
Marital status					*χ*^*2*^_(2)_ = 59.06***
Single	76.4%	83.6%	58.2%	24.2%	
Married/stable partner	12.7%	9.1%	25.5%	64.7%	
Separated/divorced	10.9%	7.3%	16.4%	11.0%	
Employment situation					*χ*^*2*^_(4)_ = 228.36***
Working	10.9%	9.1%	30.9%	96.6%	
Unemployment compensation	5.5%	3.6%	25.5%	1.1%	
On sick leave	7.3%	0%	16.4%	0%	
Disability pensión	61.8%	81.8%	12.7%	2.3%	
No income	14.5%	5.5%	14.5%	0%	
Years of schooling	9.62 ± 2.31	9.71 ± 2.15	10.38 ± 2.20	12.76 ± 1.96	*F*_(3,251)_ = 25.13***

*HC* healthy controls, *SUD* substance use disorder, *SZ* + dual schizophrenia, *SZ* schizophrenia only

***p < 0.001

Regarding clinical data (Table 2), we found differences between groups in the presence of family history of SUD, which was higher for SZ + and SUD than for SZ (*p* < 0.031 in both cases). In addition, the SZ + group showed a higher number of suicide attempts than the SUD group (*p* = 0.011), which had the lowest number. Moreover, in relation to medical disease comorbidity, as can be seen in Table 2, patients in the SUD group present more respiratory pathologies, while those in the SZ groups present more metabolic and cardiovascular pathologies (*p* < 0.01). The SUD group showed a better GAF and a lower number of prescribed psychotropic drugs (*p* < 0.001 in all cases) than the SZ and SZ + groups, with no differences between them. The intake of typical and atypical antipsychotics was higher in the two SZ groups than in the SUD group (*p* < 0.001) where only 3.6% of patients used antipsychotics. The SZ + and SZ groups differed only in the conversion to milligrams of chlorpromazine with the SZ group almost doubling that of the SZ + group (*p* < 0.001). Finally, prescription of interdictor drugs was higher for SZ + than for the SUD group (*p* = 0.049).

**Table 2:** Clinical data: schizophrenia and substance use disorder related variables

	SZ + (*n* = 55)	SZ (*n* = 55)	SUD (*n* = 55)	Contrasts
Family history of psychiatric disorders	29.1%	34.5%	21.8%	*χ*^*2*^_(1)_ = 2.20
Family history of SUD	21.8%	7.3%	29.1%	*χ*^*2*^_(1)_ = 8.68*
Medical disease comorbidity^a^				
Respiratory	9.1%	9.1%	15%	*χ*^*2*^_(1)_ = 11.72**
Metabolic	34.5%	43.6%	26.6%	*χ*^*2*^_(1)_ = 15.80**
Cardiovascular	23%	28%	12%	*χ*^*2*^_(1)_ = 13.80**
Other (HIV, epilepsy, slipped disk, rheumatism)	12.7%	14.5%	11%	*χ*^*2*^_(1)_ = 3.21
Suicide attempts	1.25 ± 1.82	0.69 ± 1.57	0.42 ± 0.90	*F*_(2,162)_ = 4.56*
GAF	63.13 ± 11.22	59.75 ± 10.15	74.50 ± 10.06	*F*_(2,162)_ = 29.52***
Number of psychiatric medications	3.30 ± 1.68	3.22 ± 1.46	0.93 ± 1.14	*F*_(2,162)_ = 47.81***
Typical antipsychotics	22.2%	25.5%	0%	*χ*^*2*^_(1)_ = 15.80***
Atypical antipsychotics	96.3%	94.5%	3.6%	*χ*^*2*^_(1)_ = 134.74***
Interdictor	37%	0%	20.0%	*χ*^*2*^_(1)_ = 24.45***
CPZ equivalent dosage (mg)	350.55 ± 281.35	617.07 ± 522.12	6.06 ± 32.13	*F*_(2,162)_ = 43.56***
SZ age of onset	23.35 ± 6.96	23.65 ± 6.71		*t*_(1,108)_ = 0.24
Duration of SZ (years)	12.65 ± 8.01	15.42 ± 9.30		t_(1,108)_ = 1.67
PANSS scores				
Positive symptoms	11.83 ± 5.70	10.30 ± 4.19		*t*_(1,108)_ = 1.46
Negative symptoms	15.58 ± 7.39	14.18 ± 7.40		*t*_(1,108)_ = 0.89
General psychopathology	31.10 ± 10.91	24.70 ± 9.01		*t*_(1,108)_ = 2.99**
SUD age of onset	17.60 ± 5.65		20.55 ± 7.24	*t*_(1,108)_ = 2.38*
Duration of SUD (years)	17.85 ± 8.03		14.61 ± 8.94	*t*_(1,108)_ = 2.00*
Number of substances used	3.62 ± 1.75		2.93 ± 1.61	*t*_(1,108)_ = 2.16*
Main substance of dependence				*χ*^*2*^_(4)_ = 6.66
Cocaine	10.9%		12.7%	
Alcohol	12.7%		9.1%	
Alcohol + Cocaine	9.1%		27.3%	
Polydrug use	67.3%		50.9%	
Type of substances used^a^				
Cocaine	92.70%		89.10%	*χ*^*2*^_(1)_ = 0.44
Alcohol	76.40%		80.00%	*χ*^2^_(1)_ = 0.21
Cannabis	76.40%		52.70%	χ^2^_(1)_ = 6.71**
Psychodysleptics	40.00%		27.30%	*χ*^*2*^_(1)_ = 1.99
Opioids	25.50%		14.50%	*χ*^*2*^_(1)_ = 2.05
Anxiolytics-hypnotics	16.40%		1.80%	*χ*^*2*^_(1)_ = 7.04**
Abstinence period (months)	6.57 ± 3.64		7.55 ± 2.61	*t*_(1,108)_ = 1.62
Number of relapses	2.25 ± 2.97		0.82 ± 1.48	*t*_(1,108)_ = 3.21**
DAST-20 (severity of addiction)	13.44 ± 2.86		13.05 ± 3.47	*t*_(1,108)_ = 0.54

Means, standard deviation, percentages, and statistical contrasts

*CPZ* chlorpromazine, *DAST-20* drug abuse screening test, *GAF* global assessment functioning, *PANSS* positive and negative syndrome scale, *SUD* substance use disorder, *SZ* + dual schizophrenia, *SZ* schizophrenia only

^a^Percentages will not equal 100 as each patient may be included in different category

*p < 0.05

**p < 0.01

***p < 0.001

Considering the clinical characteristics of the SUD, the SZ + group had an earlier age of onset (*p* = 0.019) and a longer duration of the disorder (*p* = 0.049) compared to the SUD group. In addition, the SZ + group had consumed a greater number of substances (*p* = 0.033), with no differences in the type of main substance of consumption and a majority of polydrug users in both groups. Differences were only observed in the use of cannabis (*p* = 0.010) and anxiolytics-hypnotics (*p* = 0.008) as substances, both higher in SZ + than in SUD. We found no differences between groups in the time of abstinence or in the severity of addiction, although the SZ + group presented a greater number of previous relapses than the SUD group (*p* = 0.002). Regarding the clinical characteristics of SZ, we only found differences between the SZ + and SZ groups in the general psychopathology score, which was higher in SZ + (*p* = 0.004).

### Circadian rhythms

In relation to the sleep parameters, the results revealed differences with large effect sizes between the clinical groups in the number of sleeping hours (*F*_(2,162)_ = 29.29; *p* < 0.001; *ηp*^*2*^ = 0.267) and in the time to wake-up (*F*_(2,162)_ = 18.65; *p* < 0.001; *ηp*^*2*^ = 0.188). The SUD group slept fewer hours and wake-up earlier than the SZ + and SZ groups (*p* < 0.001, in all cases). The SZ groups did not differ from each other in any sleep parameter or in the presence/absence of napping or its duration (Table 3).

**Table 3:** Sleep–wake data and circadian typology (for the clinical groups); and Distal Skin Temperature (for the clinical and control groups). Means, standard error, percentages, and statistical contrasts

	SZ + (*n* = 55)	SZ (*n* = 55)	SUD (*n* = 55)	HC (*n* = 90)	Contrasts		Significant differences (Cohen’s d)
Sleep–wake data							
Total sleep time (hours)	9.53 ± 1.77	9.71 ± 1.45	7.86 ± 0.96		*F*_(2,162)_ = 29.29***		SZ + > SUD = 1.171
							SZ > SUD = 1.505
Bed time^a^	22:54 ± 01:10	23:12 ± 01:19	23:08 ± 00:48		*F*_(2,162)_ = 2.20		
Wake-up time^a^	07:56 ± 01:45	08:34 ± 01:21	06:56 ± 00:57		*F*_(2,162)_ = 18.65***		SZ + > SUD = 0.907
							SZ > SUD = 1.882
Presence of nap	40.0%	30.9%	27.3%		*χ*^*2*^_(1)_ = 2.15		
Nap duration	81.14 ± 37.79	65.29 ± 26.49	59.00 ± 35.62		*F*_*(*2,51)_ = 2.15		
Circadian tipology					*χ*^*2*^_(2)_ = 30.31***		
Evening-type	12–7%	16.4%	3.6%				
Intermediate-type	47.3%	60.0%	20.0%				
Morning-type	40.0%	23.6%	76.4%				
CSM direct score	34.01 ± 0.87	31.28 ± 0.88	40.12 ± 0.87		*F*_(2,162)_ = 26.47***		SZ + < SUD = 0.915
							SZ < SUD = 1.208
Distal Skin Temperature					MANCOVA		
					*F*_(3,251)_ *η*_*p*_^2^		
Maximum	35.92 ± 0.07	35.91 ± 0.07	35.93 ± 0.07	36.09 ± 0.05	2.10	0.03	
Minimum	31.14 ± 0.20	31.21 ± 0.20	29.67 ± 0.20	30.56 ± 0.16	12.72***	0.13	SZ + > SUD = 1.096
							SZ > SUD = 1.190
							SUD < HC = 0.643
Mesor	33.95 ± 0.11	34.02 ± 0.11	33.15 ± 0.11	33.63 ± 0.09	13.37***	0.14	SZ + > SUD = 0.907
							SZ > SUD = 0.917
							SZ > HC = 0.514
							SUD < HC = 0.563
Acrophase^a^	01:44 ± 03:44	00:50 ± 04:20	00:58 ± 03:38	02:00 ± 02:47	1.89	0.02	
Rayleigh Vector	0.89 ± 0.03	0.96 ± 0.03	0.90 ± 0.03	0.75 ± 0.02	17.37***	0.17	SZ + > HC = 0.662
							SZ > HC = 1.461
							SUD > HC = 0.785
P1	0.60 ± 0.09	0.46 ± 0.09	0.82 ± 0.09	0.38 ± 0.07	5.51***	0.06	SZ < SUD = 0.520
							SUD > HC = 0.746
P12	1.12 ± 0.13	0.99 ± 0.13	1.83 ± 0.13	0.65 ± 0.10	17.60***	0.17	SZ + < SUD = 0.631
							SZ < SUD = 0.766
							SZ + > HC = 0.553
							SUD > HC = 1.271
CI	39.48 ± 2.84	37.86 ± 2.85	41.50 ± 2.85	49.50 ± 2.22	4.95**	0.06	SZ + < HC = 0.471
							SZ < HC = 0.567
IS	0.68 ± 0.02	0.72 ± 0.03	0.78 ± 0.02	0.39 ± 0.02	85.10***	0.55	SZ + < SUD = 0.636
							SZ + > HC = 1.702
							SZ > HC = 2.133
							SUD > HC = 2.756
IV	0.02 ± 0.33	0.05 ± 0.33	0.71 ± 0.33	0.24 ± 0.26	0.95	0.01	
RA_10	0.28 ± 0.02	0.25 ± 0.02	0.36 ± 0.02	0.23 ± 0.02	9.01***	0.10	SZ + < SUD = 0.497
							SZ < SUD = 0.701
							SUD > HC = 0.974
M5	34.94 ± 0.09	34.97 ± 0.09	34.61 ± 0.09	34.57 ± 0.07	6.13***	0.07	SZ > SUD = 0.445
							SZ + > HC = 0.606
							SZ > HC = 0.661
TM5^a^	01:37 ± 05:13	00:38 ± 04:55	01:47 ± 03:55	02:30 ± 03:16	2.12	0.03	
L10	33.19 ± 0.14	33.36 ± 0.14	32.21 ± 0.14	33.04 ± 0.11	12.45***	0.13	SZ + > SUD = 0.849
							SZ > SUD = 0.959
							SUD < HC = 0.778
TL10^a^	14:55 ± 04:37	14:06 ± 04:02	14:21 ± 04:15	14:14 ± 03:54	0.229	0.01	

*CI* circadian index, *M5* maximum mean temperature in 5 consecutive hours, *L10* minimum mean temperature in 10 consecutive hours, *IS* interdaily stability, *IV* intradaily variability, *P1* 1st harmonic power, *P12* 12th harmonic accumulated power, *RA_10* relative amplitude per 10, *SUD* substance use disorder, *SZ* + dual schizophrenia, *SZ* schizophrenia only, *TM5* maximum mean temperature time in 5 consecutive hours, *TL10* minimum mean temperature time in 10 consecutive hours

^a^Data expressed in hours and minutes (mean and standard deviation)

**p < 0.01

***p < 0.001

Regarding circadian typology, differences with large effect sizes were obtained between the groups in the mean direct CSM score (*F*_(2,162)_ = 26.47; *p* < 0.001; *ηp*^*2*^ = 0.25), being higher in the SUD group than in the SZ + and SZ groups (*p* < 0.001, in both cases), which did not differ among them (Table 3). Furthermore, we found differences in the distribution of typologies between the SUD group and the SZ + and SZ groups (*p* < 0.001, in both cases), with the SUD group having more morning-type patients and fewer evening-type patients. The SZ group has the highest proportion of evening-type patients, although without significant differences with respect to the SZ group.

**Fig. 2:**
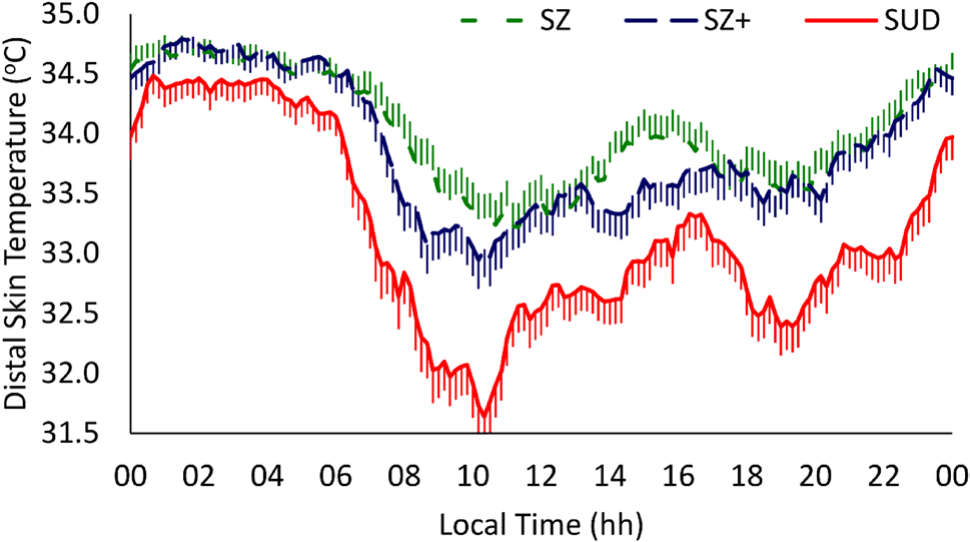
Distal skin temperature mean waveforms. Waveforms data are expressed as mean ± SEM in function of local time (hours). *SUD* substance use disorder (continuous red line, *n* = 55), *SZ* + dual schizophrenia (dashed blue line, *n* = 55), *SZ* schizophrenia only (dashed green line, *n* = 55)

Several parameters of the DST showed differences among clinical groups (see Fig. 2 and Table 3). The SUD group presented lower values with large effect sizes for minimum, mesor, and L10 with respect to the SZ + and SZ groups (*p* < 0.001, in all cases) and higher values with large medium sizes for A_10 and accumulated power of the first 12 harmonics (P12) than the other two groups (*p* ≤ 0.05, in all cases). In addition, the SUD group had higher IS than the SZ + group (*p* = 0.013), lower M5 (*p* = 0.044) and higher P1 than the SZ group (*p* = 0.029); with medium effect sizes in all cases. No significant differences were found between the SZ + and SZ groups in any of the indexes analyzed.

On the other hand, in the *posteriori* MANCOVA contrasts with the HC group (see Table 3), the SZ + group presented higher Rayleigh vector, IS, M5, and P12 (*p* < 0.023, in all cases), as well as lower CI (*p* = 0.025). Effect sizes for these mean differences were medium for Rayleigh vector, M5, and P12; and large for IS. In addition, the SZ group had higher mesor, Rayleigh vector, IS, and M5 (*p* < 0.029, in all cases) than the HC group, but lower CI (*p* = 0.006). In these cases, effect sizes were medium for mesor, M5, and IC; and large for Rayleigh vector and IS.

**Fig. 3:**
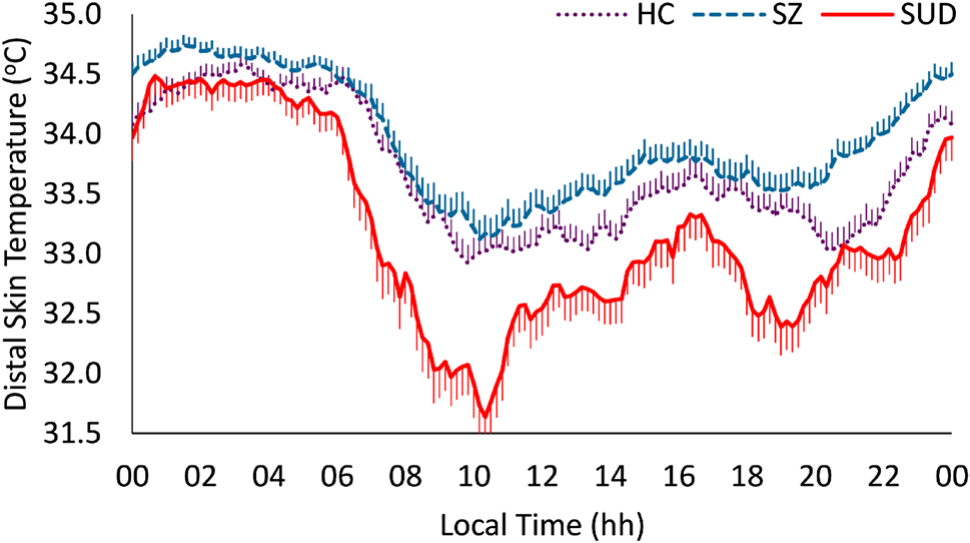
Distal skin temperature mean waveforms. Waveforms data are expressed as mean ± SEM in function of local time (hours). *HC* healthy controls (dotted purple line, *n* = 90), *SUD* substance use disorder (continuous red line, *n* = 55), *SZ* schizophrenia groups (dashed blue line, *n* = 110)

Finally, the SUD group showed lower minimum and mesor than the HC group (*p* = 0.004 in both cases), higher Rayleigh vector, P1, P12, RA_10 and IS (*p* < 0.001, in all cases) and lower L10 (*p* < 0.001) than the HC group (Fig. 3). Effect sizes for mean differences between SUD and HC groups were medium for minimum, mesor, Rayleigh vector, and P1; and large for P12, RA_10, Is, and L10 (see Table 3).

For the SZ groups considered together, we observed mixed results when compared to the HC group (Fig. 3) with higher DST values both for the sleep period and much more markedly for the waking period. This results in a lower amplitude of the rhythm and most impaired DST pattern, most marked in the SZ group (Fig. 2).

## Discussion

In the first place, no differences were observed among groups in mean age and this is relevant since circadian rhythmicity varies throughout the life cycle in sleep characteristics [34], circadian typology [9, 35], and circadian rhythm of DST [36]. Epidemiological data confirmed previous findings in similar groups [25, 37]; SZ + , SZ, and SUD diagnoses were associated to not having a partner, unemployment, disability pension, and lower level of education than HC. The SZ and SZ + groups showed a worse sociodemographic and clinical profile than the SUD group, which would confirm the presence of a greater impairment associated with SZ [4].

The groups with a diagnosis of addiction presented more family history of SUD, supporting the evidence regarding family history as a risk factor for developing the disorder [38]; the existence of family history should be considered in SUD preventive strategies. On the other hand, in line with previous studies [39, 40], the SZ + group showed earlier onset and longer duration of SUD, and greater number of substances consumed. All these characteristics are associated with worse psychopathological involvement and clinical course [41]. This underlines the importance of early problem detection and the usefulness of research for easily detectable risk factors, such as circadian disturbances (i.e. phase delay, decreased amplitude) [16]. In this sense, patients with first episodes of SZ could obtain greater benefits in the course of their disease than chronic patients such as those in our research, the consideration of the duration of untreated psychosis (DUP) being a possible clinical explanatory aspect of this.

Regarding substance use, consistent with previous studies [25, 40], most of the patients were polydrug users, although we found a higher consumption of cannabis in SZ + compared to SUD. This data is relevant since cannabis use is a risk factor for developing SZ [42]. It is also confirmed that the onset of SUD is earlier than that of SZ in the SZ + group [40, 43], emphasizing the importance of paying attention to cannabis use in adolescents/young adults, especially if they are risk population for developing a psychotic disorder. Finally, the general psychopathology score was higher in SZ + than in SZ, confirming that these patients present a worse general health status [40, 44].

Considering sleep schedules and circadian typology, the SUD group presented sleep time and duration characteristics similar to normal population data [34], and a tendency toward morningness, in line with what was expected for being under treatment. The SZ groups showed schedules and scores more typical of an intermediate-type (although in the SZ + group, there was also a greater tendency toward morningness with respect to norms), and more sleeping hours. Therefore, assessing sleep schedules and circadian typology may aid in better clinical management of both addiction [9, 45] and schizophrenia (with/without SUD). A comprehensive relapse prevention strategy could be taking these measures into account during treatment or even when changes that may affect treatment take place (i.e. withdrawal of pharmacological prescription).

The analysis of the DST pattern reflected a better circadian functioning of the SUD group with respect to SZ, SZ + , and HC groups. Thus, greater diurnal activation or less somnolence, greater endogenous control and stability were observed, similar to that previously described in patients under treatment [16, 44]. Therefore, the circadian rhythm of the SUD group has a greater day/night contrast, which would reflect a better lifestyle influenced, probably by the imposed schedule [16, 32]. In contrast, the SZ groups exhibit a DST pattern with greater rhythm stability and better nocturnal rest (greater Rayleigh vector, IS and M5) but with daytime sleepiness, which is remarkable for the SZ group, as it was previously described for sleep disordered breathing [46]. Such impairment implies a reduction in the zone of maintenance of wakefulness in the afternoon, which is associated with the presence of drowsiness, similar to the pattern observed in the elderly people [16, 47]. Likewise, the lower CI in the SZ groups may indicate a more immature circadian system [47]. These findings could be caused by alterations in thermoregulation [16, 47] or by the sedative effect of the schizophrenia medication itself [46], which should be elucidated in future studies.

The results suggest that the presence of the diagnosis of SZ is related to a worse circadian rhythmicity compared to patients with only SUD. The symptomatology of SZ favors a more sedentary lifestyle and inadequate exposure to external synchronizers [48], which has been associated with both worse rhythmic expression [23, 49] and clinical status [50]. Furthermore, circadian disturbances observed in prodromal phases of SZ points out to the possibility that these may be a key mechanism in the pathology [24, 37]. Circadian alterations in SZ contribute to poorer therapeutic outcomes [51, 52], so their evaluation and the implementation of chronobiological intervention strategies to improve rhythmic expression in these patients are of particular relevance. Thus, approaches that have proven effective in the treatment of other mental disorders [45, 53] could also be effective for SZ. These include the regular time patterns of meals, daily physical and social activity, and sleep–wakes synchronized to the light–dark cycle, without forgetting the best time to prescribe medication [54].

The study of SZ and SZ + patients indicates that rhythmic impairment is greater in the former and that it is concentrated in the wakefulness period. These data are in line with previous studies carried out in other areas, showing better premorbid functioning, executive functions, and social skills for SZ + compared to SZ. This has led to suggest that the SZ + group has a lower vulnerability for the development of SZ, although this would be modified by their substance consumption [53, 54]. Some studies suggest that the general condition of SZ + patients worsens with age, due to the cumulative neurotoxic effects of consumption [39]. Our design precludes drawing conclusions in this regard and future longitudinal research is necessary.

On the other hand, our results contrast with data about evening typology as a risk factor for the development and maintenance of SUD [9, 45]. However, research with patients under treatment and in abstinence for several months has found data similar to ours [16, 44]. The presence of a higher percentage of morning-type patients than in population norms could be interpreted as a result of the establishment in SUD treatment of healthy lifestyle habits necessary for abstinence [16, 25]. Although we do not have data on the circadian typology of the HC group, their worse DST rhythm could be due to more sedentary and delayed phase habits, in line with those results of a large part of the Spanish population, together with inadequate exposure to light both during day and night [49]. For all these reasons, our findings should be interpreted with caution. Previous studies on circadian rhythmicity in patients with SZ are scarce [55, 56] and show inconclusive results. However, DST, as a valid marker of sleep–wake rhythm and internal temporal order [12, 14], seems a sensitive measurement to differentiate between both SZ and SUD diagnoses. It is required future research evaluating DST at baseline and end of treatment, for more days (ideally a week), along with repeated measurements throughout treatment to assess the evolution and the presence of critical periods. Furthermore, since DST used alone cannot exclude masking effects produced by activity or changes in sleep, to validate the obtained results, it would be necessary to extend the assessment with more complete objective measurements, including at least light and physical activity. Moreover, the study of women in SUD and SZ + , to whom we cannot generalize our results, is urgently needed in order to provide a gender perspective in both research and treatment. All of this should be also done controlling for possible modulating factors such as medication [23] or withdrawal time [16].

## Conclusions

This study provides information not previously evaluated in any publication on circadian rhythmicity differences in under treatment male patients with diagnoses of SZ + , SZ and SUD, as well as comparing them with a HC group for DST. Our results suggest that the presence of the diagnosis of SZ (SZ + and SZ) is related to a worse circadian rhythmicity independently of the presence of a SUD. In under treatment patients with SZ, we should focus on the diurnal period assessment of circadian rhythms as possible marker of recovery, irrespective of the presence of a SUD. Our findings from male patients with a mean abstinence period longer than 6 months support the idea that the circadian rhythm of DST could be a biomarker of adherence or treatment efficacy and relapse prevention in SUD early remission, althougth these results should be confirmed with additional objective measures. Further research focused on circadian rhythmicity in patients with SZ (with/without SUD) may provide new knowledge transferable to their therapeutic management and could be a promising line to establish possible endophenotypes in the future.

## Data Availability

The data that support the findings of this study are available from the corresponding author upon reasonable request.

## References

[1] Cantor JH, Whaley CM, Stein BD, Powell D (2022). Analysis of substance use disorder treatment admissions in the US by sex and race and ethnicity before and during the COVID-19 pandemic. JAMA Net Open.

[2] Hunt GE, Large MM, Cleary M, Lai HMX, Saunders JB (2018). Prevalence of comorbid substance use in schizophrenia spectrum disorders in community and clinical settings, 1990–2017: systematic review and meta-analysis. Drug Alcohol Depend.

[3] Roncero C, García-Ullán L, Bullón A, Remón-Gallo D, Vicente-Hernández B, Álvarez A, Caldero A, Flores A, Aguilar L (2020). The relevance of dual diagnoses among drug-dependent patients with sleep disorders. J Clin Med.

[4] Río-Martínez L, Marquez-Arrico JE, Prat G, Adan A (2020). Temperament and character profile and its clinical correlates in male patients with dual schizophrenia. J Clin Med.

[5] Lynn Starr H, Bermak J, Mao L, Rodriguez S, Alphs L (2018). Comparison of longacting and oral antipsychotic treatment effects in patients with schizophrenia, comorbid substance abuse, and a history of recent incarceration: an exploratory analysis of the PRIDE study. Schizophr Res.

[6] Togay B, Noyan H, Tasdelen R, Ucok A (2015). Clinical variables associated with suicide attempts in schizophrenia before and after the first episode. Psychiatr Res.

[7] Donoghue K, Doody GA, Murray RM, Jones PB, Morgan C, Dazzan P, Hart J, Mazzonicini R, MacCabe JH (2014). Cannabis use, gender and age of onset of schizophrenia: data from the ÆSOP study. Psychiatry Res.

[8] Cambras T, Díez-Noguera A (2018). The social role of chronobiology. Biol Rhythm Res.

[9] Adan A, Archer SN, Hidalgo MP, Di Milia L, Natale V, Randler C (2012). Circadian typology: a comprehensive review. Chronobiol Int.

[10] Suh S, Yang HC, Kim N, Yu JH, Choi S, Yun CH, Shin C (2019). Chronotype differences in health behaviors and health-related quality of life: a population-based study among aged and alder adults. Behav Sleep Med.

[11] Mullington JM, Abbott SM, Carroll JE, Davis CJ, Dijk DJ, Dinges DF, Gehrman PR, Gozal D, Haack M, Lim DC, Macrea M, Pack AI, Plante DT, Teske JA, ZeePC (2016). Developing biomarker arrays predicting sleep and circadian-coupled risks to health. Sleep.

[12] Ortiz-Tudela E, Martinez-Nicolas A, Albares J, Segarra F, Campos M, Estivill E, Rol MA, Madrir JA (2014). Ambulatory circadian monitoring (ACM) based on thermometry, motor activity and body position (TAP): a comparison with polysomnography. Physiol Behav.

[13] Martinez-Nicolas A, Martinez-Madrid MJ, Almaida-Pagan PF, Bonmati-Carrion MA, Madrid JA, Rol MA (2019). Assessing chronotypes by ambulatory circadian monitoring. Front Physiol.

[14] Bonmati-Carrion MA, Middleton B, Revell V, Skene DJ, Rol MA, Madrid JA (2014). Circadian phase asessment by ambulatory monitoring in humans: correlation with dim light melatonin onset. Chronobiol Int.

[15] Prosser RA, Stowie A, Amicarelli M, Nackenoff AG, Blakely RD, Glass JD (2014). Cocaine modulates mammalian circadian clock timing by decreasing serotonin transport in the SCN. Neuroscience.

[16] Capella MM, Martinez-Nicolas A, Adan A (2018). Circadian rhythmic characteristics in men with substance use disorder under treatment. Influence of age of onset of substance use and duration of abstinence. Front Psychiatr.

[17] Laskemoen JF, Simonsen C, Büchmann C, Barrett EA, Bjella T, Lagerberg TV, Vedal TJ, Andreassen OA, Melle I, Aas M (2019). Sleep disturbances in schizophrenia spectrum and bipolar disorders—a transdiagnostic perspective. Compr Psychiatr.

[18] Løberg EM, Nygård M, Berle JØ, Johnsen E, Kroken RA, Jørgensen HA, Hugdal K (2012). An fMRI study of neuronal activation in schizophrenia patients with and without previous cannabis use. Front Psychiatr.

[19] Kaskie RE, Graziano B, Ferrarelli F (2017). Schizophrenia and sleep disorders: links, risks, and management challenges. Nat Sci Sleep.

[20] Robertson I, Cheung A, Fan X (2019). Insomnia in patients with schizophrenia: current understanding and treatment options. Prog Neuro-Psychopharmacol Biol Psychiatr.

[21] Coulon L, Brailly-Tabard S, Walter M, Tordjman S (2016). Altered circadian patterns of salivary cortisol in individuals with schizophrenia: a critical literature review. J Physiol Paris.

[22] Lamont EW, Coutu DL, Cermakian N, Boivin DB (2010). Circadian rhythms and clock genes in psychotic disorders. Isr J Psychiatry Relat Sci.

[23] Chung KF, Poon YPYP, Ng TK, Kan CK (2018). Correlates of sleep irregularity in schizophrenia. Psychiatry Res.

[24] Manoach DS, Pan JQ, Purcell SM, Stickgold R (2016). Reduced sleep spindles in schizophrenia: a treatable endophenotype that links risk genes to impaired cognition?. Biol Psychiatr.

[25] Serrano-Serrano AB, Marquez-Arrico JE, Navarro JF, Martinez-Nicolas A, Adan A (2021). Circadian characteristics in patients under treatment for substance use disorders and severe mental illness (schizophrenia, major depression and bipolar disorder). J Clin Med.

[26] American Psychiatric Association (2013). Diagnostic and statistical manual of mental disorders.

[27] Horn WT, Akerman SC, Sateia MJ (2013). Sleep in schizophrenia and substance use disorders: a review of the literature. J Dual Diag.

[28] First MB, Spitzer RL, Gibbon M, Williams JBW (2002). Structured clinical interview for DSM-IV-TR Axis I disorders, research version, patient edition (SCID-I/P). Biometrics research.

[29] Gálvez BP, Fernández LG (2010). Validación española del drug abuse screening test (DAST-20 y DAST-10). Heal Addict.

[30] Peralta V, Cuesta MJ (1994). Validación de la escala de los síndromes positivo y negativo (PANSS) en una muestra de esquizofrénicos españoles. Luso-Españolas Neurolología, Psiquiatr y Ciencias Afines.

[31] Adan A, Caci H, Prat G (2005). Reliability of the Spanish version of the composite scale of morningness. Eur Psychiatr.

[32] Martinez-Nicolas A, Madrid JA, Rol MA (2014). Day-night contrast as source of health for the human circadian system. Chronobiol Int.

[33] Sosa M, Mondéjar MT, Martinez-Nicolas A, Ortiz-Tudela E, Sarabia JA, Sosa J, Otalora B, Rol MA, Madrid JA, Campos M, Marín M. Circadianware. Spanish Patent 08/2010/183, 3 September 2010

[34] Jonasdottir SS, Minor K, Lehmann S (2021). Gender differences in nighttime sleep patterns and variability across the adult lifespan: a global-scale wearables study. Sleep.

[35] Van Someren EJW, Swaab DF, Colenda CC, Cohen W, McCall WV, Rosenquist PB (1999). Bright light therapy: improved rest-activity rhythms in Alzheimer patients by application of nonparametric methods. Chronobiol Int.

[36] Jankowski KS (2015). Composite scale of morningness: psychometric properties, validity with Munich chronotype questionnaire and age/sex differences in Poland. Eur Psychiatr.

[37] Castro J, Zanini M, Gonçalves Bda S, Coelho FM, Bressan R, Bittencourt L, Gadelha A, Brietzke E, Tufik S (2015). Circadian rest-activity rhythm in individuals at risk for psychosis and bipolar disorder. Schizophr Res.

[38] Prom-Wormley EC, Ebejer J, Dick DM, Bowers MS (2017). The genetic epidemiology of substance use disorder: a review. Drug Alcohol Depend.

[39] Benaiges I, Serra-Grabulosa JM, Adan A (2013). Neuropsychological functioning and age-related changes in schizophrenia and/or cocaine dependence. Prog Neuropsychopharmacol Biol Psychiatr.

[40] Marquez-Arrico JE, Benaiges I, Adan A (2015). Strategies to cope with treatment in substance use disorder male patients with and without schizophrenia. Psychiatry Res.

[41] Sofin Y, Danker-Hopfe H, Gooren T, Neu P (2017). Predicting inpatient detoxification outcome of alcohol and drug dependent patients: the influence of sociodemographic environment, motivation, impulsivity, and medical comorbidities. J Addict.

[42] Belbasis L, Köhler CA, Stefanis N, Stubbs B, van Os J, Vieta E, Seeman MV, Arango C, Carvahlo AF, Evangelou E (2018). Risk factors and peripheral biomarkers for schizophrenia spectrum disorders: an umbrella review of meta-analyses. Acta Psychiatr Scand.

[43] Adan A, Arredondo AY, Capella MD, Prat G, Forero DA, Navarro JF (2017). Neurobiological underpinnings and modulating factors in schizophrenia spectrum disorders with a comorbid substance use disorder: a systematic review. Neurosci Biobehav Rev.

[44] Antúnez JM, Capella MM, Navarro JF, Adan A (2016). Circadian rhythmicity in substance use disorder male patients with and without comorbid depression under ambulatory and therapeutic community treatment. Chronobiol Int.

[45] Adan A (2013). A chronobiological approach to addiction. J Subst Use.

[46] Martinez-Nicolas A, Guaita M, Santamaría J, Montserrat JM, Rol MA, Madrid JA (2017). Circadian impairment of distal skin temperature rhythm in patients with sleep-disordered breathing: the effect of CPAP. Sleep.

[47] Batinga H, Martinez-Nicolas A, Zornoza-Moreno M, Sánchez-Solis M, Larqué E, Mondéjar MT, Moreno-Casbas M, García FJ, Campos M, Rol MA, Madrid JA (2015). Ontogeny and aging of the distal skin temperature rhythm in humans. Age.

[48] Scheewe TW, Jörg F, Takken T, Deenik J, Vancampfort D, Backx FJG, Cahn W (2019). Low physical activity and cardiorespiratory fitness in people with schizophrenia: a comparison with matched healthy controls and associations with mental and physical health. Front Psychiatr.

[49] Martinez-Nicolas A, Madrid JA, García FJ, Campos M, Moreno-Casbas MT, Almaida-Pagán PF, Lucas-Sánchez PF, Rol MA (2018). Circadian monitoring as an aging predictor. Sci Rep.

[50] Lunsford-Avery JR, Gonçalves BDSB, Brietzke E, Bressan RA, Gadelha A, Auerbacj RP, Mittal VA (2017). Adolescents at clinical-high risk for psychosis: circadian rhythm disturbances predict worsened prognosis at 1-year follow-up. Schizophr Res.

[51] Mulligan LD, Haddock G, Emsley R, Neil ST, Kyle SD (2016). High resolution examination of the role of sleep disturbance in predicting functioning and psychotic symptoms in schizophrenia: a novel experience sampling study. J Abnorm Psychol.

[52] Li SX, Lam SP, Zhang J, Yu MW, Chan JW, Chan CS, Espie CA, Freeman D, Mason O, Wing YK (2016). Sleep disturbances and suicide risk in an 8-year longitudinal study of schizophrenia-spectrum disorders. Sleep.

[53] Kragh M, Martiny K, Videbech P, Møller DN, Wihlborg CS, Lindhardt T, Larsen ER (2017). Wake and light therapy for moderate-to-severe depression—a randomized controlled trial. Acta Psychiatr Scand.

[54] Koizumi T, Suzuki T, Pillai NS, Bies RR, Takeuchi H, Yoshimura K, Mimura M, Uchida H (2019). Circadian patterns of hallucinatory experiences in patients with schizophrenia: potentials for chrono-pharmacology. J Psychiatr Res.

[55] Ahn YM, Chang J, Joo YH, Kim SC, Lee KY (2008). Kim YS (2008) chronotype distribution in bipolar I disorder and schizophrenia in a Korean sample. Bipolar Disord.

[56] Thomas P, He F, Mazumdar S, Wood J, Bhatia T, Gur RC, Gur RE, Buysse D, Nimgaonkar VL, Deshpande SN (2018). Joint analysis of cognitive and circadian variation in schizophrenia and bipolar I disorder. Asian J Psychiatr.

